# Coping strategies and considerations regarding low anterior resection syndrome and quality of life among patients with rectal cancer; a qualitative interview study

**DOI:** 10.3389/fonc.2022.1040462

**Published:** 2022-11-29

**Authors:** Birgitte Schantz Laursen, Gitte Kjær Sørensen, Margit Majgaard, Line Byskov Jensen, Karen Irene Jacobsen, Dorte Kløve Kjær, Therese Juul, Peter Christensen, Anette Højer Mikkelsen

**Affiliations:** ^1^ Sexology Centre and Gastrointestinal Surgical Outpatient Clinic, Aalborg University Hospital, Aalborg, Denmark; ^2^ Department of Clinical Medicine, Aalborg University, Aalborg, Denmark; ^3^ Department of Surgery, Aarhus University Hospital, Aarhus, Denmark

**Keywords:** low anterior resection syndrome, major LARS, quality of life, coping strategies, qualitative study, qualitative interviews, thematic analysis

## Abstract

**Introduction:**

Low anterior resection syndrome (LARS) is defined as disordered bowel function following rectal resection, which is detrimental to quality of life (QoL). A recent international consensus definition of LARS stresses the importance of focusing on both the symptoms and the consequences that the symptoms have for the individual patient as studies indicate that LARS has a negative impact on patients’ QoL. However, an ongoing PROM study investigating late sequelae after rectal cancer finds that a minor proportion of patients scoring major LARS experience none or only little impact on quality of life

**Aim:**

The aim of this study was to identify patients’ considerations and coping strategies to establish why the burden caused by major LARS had little or no influence on their QoL.

**Materials and methods:**

This was a qualitative interview study based on 21 semi-structured individual telephone interviews with patients treated for rectal cancer. Data were analysed using a hermeneutic inspired thematic analysis.

**Results and conclusion:**

Three themes emerged from the analysis; Adapting new life situation, Altering life perception and the Importance of relationships. Major LARS and its consequences following rectal cancer may be managed or altered by adopting problem-focused and emotion-focused coping strategies. Maintaining a positive attitude and having a good network of family and friends constitute a surplus, allowing patients to cope with the need for changed behaviour and appreciate the life that they have been given. Accepting that major LARS and its consequences cause limitations in life allowed patients to change their normality threshold over time.

## 1 Introduction

The prevalence of cancer in the Northwest European adult population is estimated at 4.4%, and the overall survival rate of cancer patients has increased in recent decades owing to improved treatment modalities (1). Every year, 45,000 Danes are diagnosed with cancer, and there are about 365,000 survivors of cancer treatment in Denmark (2). One logical consequence of the increased survival rate is a shift in focus from biomedical therapeutic procedures to improving survivorship skills and quality of life (QoL). Attention to late sequelae after cancer has, therefore, risen.

Worldwide, colorectal cancer is one of the most predominant cancers, representing 10.9% of all cancers in males and 9.5% in females (3). A common late sequela following a low anterior resection (LAR) for rectal cancer is the LAR syndrome (LARS), which is pragmatically defined as disordered bowel function after LAR leading to a deterioration of QoL (4). A recent international consensus definition of LARS stresses that it is of great importance to focus both on the symptoms and the consequences that the symptoms have for the individual patient (5). Studies have indicated that LARS has a negative impact on patients’ QoL in up to 80% of cases with major alterations in 40%. Still, a recent study found that from a clinical viewpoint, the burden caused by LARS on the QoL of patients treated for low and mid rectal cancer is frequently underestimated (6). However, an ongoing patient-reported outcome measures (PROM) study investigating late sequelae after rectal cancer found that some patients scored none or only little impact of major LARS on their QoL. The inconsistent correlations between symptoms measured by the LARS score and QoL could be caused by limitations of the LARS score, and it would therefore be of interest to see how patient’s perspectives and coping strategies interact with their LARS to impact on their QoL. Understanding the mechanism behind and getting insight into the perspectives and the coping strategies used by patients who experience no or little impact on their QoL despite major bowel dysfunction may be used by healthcare professionals to support and guide patients who have major LARS but experience great impact on their QoL.

Thus, the aim of this qualitative study was to identify patients’ considerations and coping strategies explaining why the burden of major LARS had potentially little or none influence on their QoL.

## 2 Methods

The study design was qualitative, and the study was conducted under the Danish Cancer Society Centre for Research on Survivorship and Late Adverse Effects after Cancer in the Pelvic Organs (7). The study was based on 21 individual semi-structured telephone interviews with patients undergoing LAR for rectal cancer and experiencing LARS symptoms. The applied interpretive data-driven thematic analysis gives voice to patients, which is useful when focusing on patient experiences (8). The study was reported following the Consolidated Criteria for Reporting of Qualitative Research (COREQ) (9).

### 2.1 Participants

The participants were recruited from the study “Systematic screening for late sequelae after colorectal cancer” initiated by the Danish Cancer Society Centre for Research on Survivorship and Late Adverse Effects after Cancer in the Pelvic Organs. In the study, patients with colorectal cancer (CRC) complete questionnaires at 3, 12, 24 and 36 months after surgery. The selected domains in the questionnaire include bowel, urinary and sexual dysfunction, chronic pain and stoma problems (10). The participants included in the present study had a LAR for rectal cancer with or without chemo-/radiotherapy, and they had no stoma at the time of the interview. Participants who previously had a temporary diverting stoma had it reversed a minimum of six months prior to the interview. All participants had completed the PROMs and scored major LARS, but at the same time they stated that their bowel function had no or only little impact on their QoL. The patients were invited to participate in the present study consecutively between one and three years after surgery. A total of 21 patients were included in the study to ensure data saturation (11).

#### 2.1.1 Inclusion criteria

The sampling was purposeful and criterion based.

1. Rectal cancer patients undergoing a LAR, without a present stoma, included in the study “Systematic screening for late sequelae after colorectal cancer”,
2. Patients who scored major LARS and experienced no or only little impact of bowel function on QoL.

Participant characteristics are presented in **Table 1**.

**Table 1:** Patient characteristics.

Patient id	Age	SexM/F	LARS Score	Impact on Quality of life*	Work situation	Civil status	Previous stoma	Month since operation
1	74	M	37	a little	retired	married	yes	12
2	54	M	34	a little	working	married	yes	12
3	72	M	31	a little	retired	married	no	12
4	66	F	33	a little	retired	married	yes	12
5	72	M	33	a little	retired	married	yes	12
6	65	M	37	a little	retired	married	no	3
7	62	F	36	a little	working	married	yes	24
8	70	F	37	a little	retired	married	no	3
9	63	M	36	a little	working	married	no	24
10	84	F	37	not at all	retired	married	yes	12
11	59	M	39	a little	retired	single	no	12
12	63	F	36	a little	working	married	no	24
13	60	M	35	a little	working	married	yes	12
14	50	F	36	a little	working	married	yes	12
15	68	M	35	a little	retired	married	yes	12
16	60	F	37	a little	working	married	yes	12
17	54	F	37	a little	working	married	yes	12
18	73	F	31	a little	retired	widow	yes	12
19	73	F	39	a little	retired	married	yes	12
20	75	M	34	a little	retired	married	yes	12
21	50	F	36	a little	working	married	yes	12

*Question: overall, how much does your bowel function affect your quality og life? Options for answering not at all, a little, some, a lot.

### 2.2 Data collection

The telephone interviews were conducted by authors 2-5 who all are registered nurses, based on a semi-structured interview guide, **Table 2**, with open questions in line with the aim of the project and research in the field (9). As an introduction, the participants were asked to present themselves and their experiences during their disease course and treatment. The interview focused on the patients’perspectives, experiences and thoughts in relation to their bowel dysfunction and its impact on their QoL (**Table 2**).

**Table 2:** Interview guide.

Research question	Interview theme
**How patients manage their LARS symptoms in everyday life. Despite LARS, why is their QoL not affected?**	**We can see from your answer to the questionnaire that your stool pattern has changed, what does a normal day look like for you?**
	**How do you adjust to your bowel dysfunction? (Has anything in the house decor, diets, etc., changed)?**
	**How does your bowel dysfunction affect your day and your thoughts?**
**Why do the patients’ LARS symptoms NOT affect his/her QoL and life development?**	**Have you stopped doing things because of your bowel dysfunction that you did in the past? (holidays, cinema visits, restaurant visits, visiting friends and family, walking, shopping, etc.)?**
	**What makes you think you have a good life?**

The individual interviews allowed participants to raise topics and express thoughts that they considered important. The interviews were audiotaped and lasted 15-30 minutes. Data were collected from October 2020 to June 2021 and all interviews were transcribed.

### 2.3 Data analysis

Data were analysed collaboratively by the authors. To search for meaningful patterns (themes) across the interviews, an inductive, data-driven thematic analysis was conducted (8). The interpretation of the interviews was initiated by transcription of the verbal data, obtaining an overview of all the interviews focusing on the patients’ perspectives, experiences and thoughts. Then, more structured and analytically meaningful themes and patterns were identified, defined and named. In the final phase, the themes were interpreted and discussed in relation to other research and theories in the explored field.

### 2.4 Ethical considerations

Ethical considerations followed the directions of the Helsinki Declaration. All participants were informed, and confidentiality was ensured. Recommended procedures to ensure informed consent and voluntariness were followed (7). The study was reported to and approved by the Danish Data Protection Agency (no. 2019-110). Data were anonymized using numbers and was stored securely.

## 3 Results

Three themes emerged from the narratives shared by the patients on their experiences living and coping with LARS following rectal cancer. The themes were: Adapting to a new life situation; Altering life perception and Importance of relationships.

### 3.1 Adapting to a new life situation

All the patients had changed various aspects of their everyday life to cope with the changes introduced due to bowel dysfunction. They had become more observant of how their body and their bowel movements reacted to diet, activities and medication, which had allowed them to plan their lives so that the disease affected them as little as possible. In general, patients had accepted and learned to live with their bowel problems: “I have learned to adapt”. (13)

One of the men explained how increased attention to his body’s signals helped him to control his defecation:

   “Well, I actually think that I have become a bit better, you know, at sensing when I need to go … and, usually, I can feel that I have finished … but occasionally I feel nothing and that’s when things get messy (involuntary bowel movements)”. (17)

One of the women had trained her pelvic floor, allowing her to better control her bowel movements:

   “Well, I attended rehabilitation and learned how to train my pelvis … I think that helps me keep my bowels back for longer… (19)

Another man described how he dealt with his increased flatus:

   “I’m not bothered by it, I just sit there, real quiet, and lean to one side for a moment to let out a bit of gas. Usually, It’s silent or the sound is so low that it doesn’t matter, even though you are with other people”. (20)

Most patients described how, over time, they had become aware of how their diet and fluids affected their bowel function. Based on their observations, they had adopted individual strategies so that their daily lives were less affected by their bowel dysfunction. These strategies comprised the ingredients in the food and the quantity of food ingested.

    “I used to just dig in when I was enjoying a meal in good company, I just kept eating, I don’t do that anymore because when I do I feel like shit the next day”. (9)

Not all patients refrained from having their favorite dishes. They arranged themselves and in cases in which they knew from experience that a certain type of food would affect their bowel movements, they made sure that they were close to a toilet.

    “It’s because I know how to tackle it, right. I think that I know how to handle it really well. I shouldn’t start out by having beans or cabbage or something like that if I know that I’ll be going out later in the evening (laughs), because then you never know what might happen. Let’s say that we decide to have a nice lunch with some schnapps and beer, then we’ll do so at home where I can get to the toilet without delay”. (1)

If the patients knew that they might not have access to a toilet, some of them chose to use a diaper as security.

   “Let’s say that I’m going out and that I’m unsure if there’s a toilet close by. Well, then I’ll just put on a diaper. And that will allow me to feel safe”. (1)

Other patients regulated their bowel movements by adjusting their food and drink intake.

   “I need to plan ahead if I’m going travelling … ehh … then I’ll fast until I reach my destination, mostly because then I don’t need to make sure that a toilet is close by”. (7)

In addition to considering the amount of food and when to consume it, patients had also figured out which foods to avoid not to experience intestinal problems.

   “I’ve learned things like avoiding spicy food. Basically, food shouldn’t be too spicy or fatty and onion will also get you into trouble. All of the things that typically get your intestines going, I’m more sensitive to those now.” (13)

Patients occasionally used medication to control their bowel function; medication that stopped or promoted bowel movements. A single patient utilized the side effects of morphine to gain a night’s rest:

   “And I take 5 or 10 milligrams of morphine every night to get a good night’s sleep. I don’t take it to manage pain, it’s to calm my peristalsis”. (9)

A patient explained that when he could feel that he needed to go to the toilet 3-4 times within a short period of time, he took medication to slow down his bowel function:

   “Once in a while, I don’t get to the toilet in time and then things get messy; sometimes I have to take off towards the toilet up to four times … then I take one of the stop pills that I got at the hospital; otherwise I can’t handle it”. (17)

One of the interviewed males experienced that bowel dysfunction prevented him from activities that he could before, and this had added to his QoL:

   “I’m a bit of a nature freak … I find that to be quality of life. I used to go hunting a lot and hiking and sleeping in shelters and stuff like that. You just don’t do that anymore”. (2)

### 3.2 Altering life perception

A person’s basic perception or attitude towards life is typically reflected in his or her behaviour and thoughts. In the present study, the patients’ life attitude played a considerable role in determining how they dealt with their bowel problems and the ensuing changes. They had accepted their new living conditions, had chosen to adopt a positive outlook on life and were thankful that they were alive. In that context, the bowel problems were not allowed to take over.

One of the men had put it as follows:

   “But then, I say, you know what, if my gut problems are what allows me to stay alive then I can live with it. Sure, it can be annoying and some days are worse than others, but I take that in my stride”. (3)

Another patient strategy was to compare themselves with others and find that they felt significantly better than some of the patients they met at the hospital or in the surrounding community or that their situation was far better than theirs.

For some of the patients, it was important that people surrounding them could not see that they were ill; this meant that they were not constantly reminded that they had been ill and now had bowel dysfunction. One of the patients expressed this as follows:

   “You know what, nobody can see that I’m ill, and that is a good thing because no one is looking at me and talking about me. There’s a young girl in town, 19 years old, I think. She has lost a foot to cancer and everybody can see that”. (14)

In addition, most patients had a stoma immediately after their surgery and nearly all patients found that their current intestinal problems were preferable to the problems they had experienced with the stoma:

   “I’m glad I got rid of the stoma. That was no fun at all, definitely not. I couldn’t keep any food in me. It all passed right through, and I lost a lot of fluids if I ate stuff I weren’t supposed to … I figured out that bananas and spicy buns worked. Things went better If I only had those. Now, I think, I’m nearly normal” (4)

The same patient compared her current situation to her stoma period, noting:

   “If I had still had the stoma, I would have had problems, or it would have affected my quality of life more “. (4)

Not neglecting or repressing the problems you encounter in life and being open to family and friends and involving them in the problems helped patients cope the challenges that their gut problems presented them with.

One patient explained that letting his surroundings know how he feels made him feel better:

   “That everyone sort of knows how I feel, it’s important for me to be open about it and, like, just talk about it, so that people around me don’t feel uncomfortable and that they might say something inappropriate”. (17)

Having an open approach to the problems you experience in life and being open to the people surrounding you so that they know how you feel helped patients deal with the challenges that their bowel problems presented.

How one’s psyche is and how one generally tackles life’s challenges is also evident in situations where one has to learn to live with the late effects of chronic illness.

A man explained:

   “Generally, I think I’m a tolerant guy. I get used to lots of stuff, right? That’s just the way it is. But I have, as I said before, I have chosen that this would not bother me. It would not control my life, so I just try to make things work”. (2)

Another man noted:

    “But I think, I can’t do anything about it anyway. So why the heck should I feel sad and angry and blue about it”. (3)

### 3.3 Importance of relationships

Having a good network was very important when you are affected by an illness and have to live with late effects that affect your life. Experiencing support from family and friends meant for many that the illnesses became easier to deal with.

   “Well, I can only say that I have a lovely life as a senior citizen, I enjoy spending time with my wife and we have fun together. That’s quality of life. We have many friends and they visit us and we visit them. I don’t let it [the late effects] affect my life”. (20)

Another woman added:

   “It’s important to be enjoying your marriage and that you have a strong relationship with your children and grandchildren, I’d say. That would be it”. (10)

A good close and intimate relation was very important for most of the patients:

   “Well, I have a loving husband and all, and we’re enjoying life and have sex. So things are working out fine”. (8)

Not only partners were important; so were supportive good friends:

   “And I enjoy going to the beach, along the waterfront, just taking it all in. Sometimes I bring a friend. Then we go for a long walk and talk about important things in our lives”. (8)

## 4 Discussion

To our knowledge, this is the first study identifying coping strategies and considerations that facilitate living a nearly normal life despite cancer experience, symptoms and their long-term consequences for daily life. The themes identified in this study were: adapting to a new life situation; altering life perception; and the significance of relationships. These three coping strategies, some of which were developed during the patients’ cancer experience, helped them integrate their cancer experience into their everyday lives and face the physical and psychosocial challenges arising from cancer, and allowed them to live their lives as normally as possible.

The ability to find meaning and coherence in life is related to the ability to cope with the stressors to which we are exposed. According to the Israeli sociologist Antonovsky’s qualitative concept “sense of coherence”, finding meaning in life is associated with feelings of comprehensibility, manageability and meaningfulness. To achieve a strong sense of coherence presupposes that the person experiences predictability, stress balance and a measure of influence on his or her life situation (12). In the present study, bowel dysfunction may be defined as a stressor. To achieve a strong sense of coherence, it is of essential importance that the patients are able to cope with the external symptoms and their changed life situations. However, humans’ ability to deal with stressful challenges is closely linked to their life attitudes and ability to apply coping strategies.

According to Lazarus and Folkman, humans have two basic coping strategies, problem-focused and emotion-focused coping, as responses aimed at “managing or altering the problem causing distress” and “regulating emotional responses to the problem,” respectively (13). The former focus on solving or processing problems, expanding action options, seeking information or confrontation. In contrast, the latter focus on regulating emotions and discomfort and mentally shifting focus or seeking comfort/relief. (Ibid).

The patients in this study applied both problem- and emotion-focused coping. They adopted a problem-focused strategy by which they actively aimed to address the challenges that their LARS symptoms presented them with. Several of the patients had exercised an awareness of their body and had been able to sense the signals from the intestinal system so that they could be eliminated in situations of willing bowel movements, and one says that she exercised the pelvic floor to improve control over her bowel movements. Another problem-focused strategy was to adjust the diet to limit bowel problems. A single patient found that if he took morphine, which reduces intestinal peristalsis, he could avoid having to run to the toilet. Some patients accepted that the bowel dysfunction had introduced limitations into their lives and had thus changed their normality threshold over time. This is in line with findings in a study by Bohlok et al. who found no correlation between overall QoL and LARS and argued that this might be due to the patient’s ability to accept and adjust to their new life situation (6). More than half of the patients in the present study were retired, which also made it easier for them to plan their day according to their bowel dysfunction.

In relation to the emotion-focused strategy, patients focused on understanding the emotions and discomfort associated with LARS by shifting their focus and using their network. One strategy used to shift the focus was to compare their own current situation with a previous situation that they felt was much more serious. Half of the patients initially had a temporary stoma which was subsequently reversed, and some of them experienced that the time with the stoma involved far more challenges and discomfort, which had a positive impact on their perception of life with LARS.

Other patients compared their current situation to that of others, thereby putting their experiences into perspective and reaching the conclusion that they were much better off than some others. One of the patients compared his own situation with that of a 19-year-old girl who has had her leg amputated due to cancer and concluded that her situation was “far worse”. Scaling your own experience and measuring your suffering against that of others is coined “response shift” and has also been observed as a coping strategy in other studies (14, 15)

Having a good family and a strong network helped patients cope with the situation. Studies have shown that partners constitute a particularly important emotional support, which is directly associated with a higher mental and physical health related QoL (16). Haviland et al. concluded that poorer social support is significantly associated with a poorer health related QoL in colorectal cancer (17). Similarly, a recent questionnaire survey among Danish cancer survivors found that support from a relative was the most important factor in overcoming a cancer course (18).

Family and friends can provide support by being present and by listening to the patient, but they may also help divert attention from cancer and shift the patient’s focus to things in life that are of great importance. Suffering decreases when friends and family members take an active part in the disease course, not only during the acute phase but also in the subsequent period. This is supported by a study (19) arguing that suffering must be understood in a social context. If the surroundings do not understand the importance of the patient’s suffering, the patient must bear the suffering alone, which may aggravate the process by adding a feeling of loneliness (19). Studies show that loneliness may lead to greater depressive symptoms and poorer QoL than in patients who experience no loneliness (20, 21).

Experiencing joy of life gives courage to live and promotes the unfolding of life, helping the patients to feel less inhibited and limited by their bowel dysfunction and the ensuing consequences. People who adopt a positive attitude, feel more joy and can achieve a strong sense of coherence and self-care ability (22).

Limitations of the study is according to data saturation as it is not possible to achieve a 100% data saturation. Furthermore, the homogeneity of the sample with regard to ethical and geographical representation may limit the generalizability or findings to more diverse population. Moreover patients’ considerations and coping strategies might be reducing the negative impact on QoL in major LARS, but it could also be at play in minor LARS therefore the causal relationship requires further study, as treatments, medication etc. also can explain reduced impact on QoL.

## 5 Conclusion

Bowel dysfunction and its consequences after rectal cancer may be managed or improved by using both problem-focused and emotion-focused coping strategies. Being able to alter life perception and having a good network of family and friends produces a surplus allowing patients to adapt to the need for changed behaviour and to appreciate the life that they have been given. Accepting that bowel dysfunction and its consequences come with limitations in life has allowed the patients to change their normality threshold over time.

## Data availability statement

The original contributions presented in the study are included in the article/supplementary material. Further inquiries can be directed to the corresponding author.

## Ethics statement

Ethical review and approval was not required for the study on human participants in accordance with the local legislation and institutional requirements. The patients/participants provided their written informed consent to participate in this study.

## Author contributions

Conceptualization: BL, GS, MM, LJ, KJ, DK, TJ, PC, and AM. Methodology: BL and AM. Data collection: GS, MM, LJ, KJ, and DK. Data analysis: BL, GS, MM, LJ, KJ, DK, and AM. Preparation of the original draft: BL. Writing, review, and editing: BL, GS, MM, LJ, KJ, DK, TJ, PC, and AM. All authors contributed to the article and approved the submitted version.

## Funding

The study was supported by a grant from the Danish Cancer Society (Grant number R192-A11536).

## Acknowledgments

The authors take this opportunity to express their gratitude to the patients who have contributed their stories and experiences thereby making this study possible.

## Conflict of interest

The authors declare that the research was conducted in the absence of any commercial or financial relationships that could be construed as a potential conflict of interest.

The reviewer CV declared a past collaboration with the author PC to the handling editor.

## Publisher’s note

All claims expressed in this article are solely those of the authors and do not necessarily represent those of their affiliated organizations, or those of the publisher, the editors and the reviewers. Any product that may be evaluated in this article, or claim that may be made by its manufacturer, is not guaranteed or endorsed by the publisher.
